# Molecular Dynamics Study on the Mechanism of Gallium Nitride Radiation Damage by Alpha Particles

**DOI:** 10.3390/ma16124224

**Published:** 2023-06-07

**Authors:** Yang Liu, Zhenpeng Xiong, Xiaoping Ouyang

**Affiliations:** 1 School of Nuclear Science and Engineering, North China Electric Power University, Beijing 102206, China; 2 State Key Laboratory of Nuclear Resources and Environment, East China University of Technology, Nanchang 330013, China; 3 State Key Laboratory of Intense Pulsed Radiation Simulation and Effect, Xi’an 710024, China

**Keywords:** molecular dynamics, GaN, α-particle, displacement damage effects, cumulative injection, amorphization

## Abstract

In special applications in nuclear reactors and deep space environments, gallium nitride detectors are subject to irradiation by α-particles. Therefore, this work aims to explore the mechanism of the property change of GaN material, which is closely related to the application of semiconductor materials in detectors. This study applied molecular dynamics methods to the displacement damage of GaN under α-particle irradiation. A single α-particle-induced cascade collision at two incident energies (0.1 and 0.5 MeV) and multiple α-particle injections (by five and ten incident α-particles with injection doses of 2 × 10^12^ and 4 × 10^12^ ions/cm^2^, respectively) at room temperature (300 K) were simulated by LAMMPS code. The results show that the recombination efficiency of the material is about 32% under 0.1 MeV, and most of the defect clusters are located within 125 Å, while the recombination efficiency of 0.5 MeV is about 26%, and most of the defect clusters are outside 125 Å. However, under multiple α-particle injections, the material structure changes, the amorphous regions become larger and more numerous, the proportion of amorphous area is about 27.3% to 31.9%, while the material’s self-repair ability is mostly exhausted.

## 1. Introduction

In the future, deep space exploration will require multi-functional electronic devices [1,2]. As one of the cornerstones of the modern electronics industry, semiconductor materials have undergone a long development process. With significant improvements in manufacturing technology and application areas, researchers have further studied and developed third-generation semiconductors based on second-generation semiconductors, such as silicon carbide (SiC) and gallium nitride (GaN). Third-generation semiconductors have higher performance, lower power consumption, and broader application fields than the first two generations of semiconductors. Therefore, third-generation semiconductors are considered the cutting edge of semiconductor technology. Gallium nitride semiconductor materials, as essential third-generation semiconductor materials, have a large bandgap (3.1 eV), a stable high breakdown field (3.5 × 10^6^ V/m), high electron saturation velocity (2 MV/cm), and high thermal conductivity (1.3 W/cm·K). As a result, they are widely used in modern electronic devices, detectors, and other fields. In addition, gallium nitride also has excellent heat- and radiation-resistant characteristics, making it widely applicable in military and aerospace domains [2,3,4].

When the material is working in a harshly irradiated environment, radiation will result in severe degradation of material properties. Many studies have observed this phenomenon in different materials [5,6]. Radiation particles interact with semiconductor materials, mainly through nuclear scattering or nuclear reactions, which deposit a large amount of energy within the semiconductor material and result in the deposition of recoil atoms or reaction products inside the material [7]. Nuclear energy development has two main types: nuclear fusion energy and nuclear fission energy. Compared to nuclear fission reactions, nuclear fusion produces many α-particles. As a third-generation semiconductor detector, studying the radiation damage of GaN caused by α-particles is an essential topic in semiconductor development. Using α-particles to irradiate semiconductor materials directly is the most direct and effective method to study radiation damage effects, and a large number of semiconductor materials irradiated by α-particles have been investigated through various domestic and international experimental reactors, particle accelerators, or other radioactive sources, accumulating a wealth of experimental data. Although current experimental equipment can observe or identify specific defects in semiconductors, it has certain limitations in observing the entire irradiation process [8]. Therefore, relying solely on irradiation experiments cannot simulate every detail of the interaction between particles and matter, nor can it be used to analyze the causes of these defects. Moreover, the lengthy experimental cycles and high costs pose significant research barriers. In addition to experiments, the interaction between particles and matter can be simulated by computer. At present many computer codes have been developed to study the radiation damage. However, due to the limitation of computing power, the more detailed the calculation results, the smaller the calculation scale. In this work, the molecular dynamics method is used, which can simulate the damage effect of large-scale irradiation at the molecular level, and can clearly observe the damage-generation process.

In recent years, many scientists have used computer simulations to study the radiation damage to materials. He et al. [7] studied the displacement damage of GaN after 1, 10, 100, and 500 MeV proton irradiations. They reported that the NIEL deposited per unit thickness decreased with increasing proton energy. Li [9] found that 30 keV helium ions injected into monocrystalline silicon would produce dislocation, and the energy level and band structure would be affected. He et al. [8,10] simulated Ga PKA bombardment of the GaN matrix at 300 K to 900 K and found that the displacement threshold energy of GaN increases with the increase of temperature, and the defect trend is similar, but the higher the temperature, the higher the recombination efficiency. Xie et al. [11] used the Monte Carlo method to study neutron irradiation of GaN under different irradiation environments. The results showed that the PKA energy was lower, and the distribution range was wider under atmospheric neutron spectra. Ullah et al. [12,13,14] studied the defect production process in GaN under 50 eV/amu single atomic and molecular ion irradiation. The study showed that the damage varied nonlinearly with increasing molecular weight. Khanal et al. [15] conducted proton irradiation experiments on GaN at 100 KeV and found that the device performance decreased with increasing proton flux. Kucheyev et al. [16,17] studied the cascade density of GaN under keV cluster ion bombardment at room temperature and liquid nitrogen temperature. The study showed that the cascade density increased with increasing temperature, and the damage efficiency of molecular ion irradiation of GaN was higher than that of its single constituent ions. Karaseov et al. [18] used MD simulations to study the formation of damage caused by P, Ag, and PF4 ions on GaN. The results showed that the damage caused by Ag was more significant, and the large defect clusters increased nonlinearly. Chen et al. [19] studied the production and evolution of defects in GaN under 500 eV–40 KeV PKA irradiation. The study showed that many atoms would undergo displacement in a compact cascade volume during the collision stage under proton irradiation. Still, a significant combination of vacancies co-occurred, called pseudo-metallic behavior (PMB). Hosseini et al. [20] used MD simulations to study the defect and temperature effects of Ar atom bombardment on GaN. The results showed that as the temperature increased from 300 K to 350 K, the atomic loss in GaN increased, and the atomic mechanical stability decreased. Li [21] used molecular dynamics simulations to study the injection of α-particles into single-crystal silicon, and the results showed that a dislocation structure was produced during the injection process. Fan et al. [22] used Monte Carlo calculations to simulate the irradiation of aluminum materials by protons and α-particles. The results showed that the damage caused by α-particles was more significant than that caused by protons. Pan et al. [23] used computer simulations to study the radiation damage effects of fusion α-particle bombardment on amorphous targets (diamond and iron targets). It was concluded that the peak of elastic energy deposition was 5 eV/Å in both targets. The number of vacancies was proportional to the elastic energy deposition and inversely proportional to the displacement threshold energy. Li et al. [24] simulated the epitaxial growth of GaN films at different particle incident energies. The results show that increasing the incident energy of particles can improve the epitaxial surface mass.

Molecular dynamics simulation (MD) is a popular and reliable computer simulation method that can describe molecules’ motion at the microscopic level. Recently, MD simulation has been used to calculate the atomic/mechanical properties of materials [25,26,27,28,29]. In addition to the MD methods described above, ab initio molecular dynamics studies (AIMD) have also been widely used in recent years and have been successfully applied to group IIIA nitrides and group IIIA binaries [30,31]. All these approaches have become popular recently and will become important in the future. Therefore, this article uses molecular dynamics simulation to study the radiation damage mechanism of α-particles on GaN materials, which can better understand the evolution of damage inside the material during collision cascades and further understand the damage mechanism of α-particle irradiation on GaN. The innovation point of this work is that the GaN model is an imperfect matrix with vacancy defects and doped Fe atoms. Secondly, the α-particle is selected as the incident particle, and the single incident particle is compared with the cumulative incident particle. This work is of great significance for studying the irradiation effects of GaN-based devices in space radiation environments or nuclear fusion reactor environments.

## 2. Simulation Method

The MD simulation method can be used to simulate the process of material defect generation [19]. In this article, all simulations were performed using the open-source parallel MD code LAMMPS, which has been used to simulate the radiation damage of materials [32,33,34]. The results were analyzed using the visualization software OVITO [35,36,37]. At room temperature, GaN consistently exhibits the crystal structure of wurtzite, which is a hexagonal crystal system. The lattice constants of wurtzite GaN are a=b=3.216 Å and c=5.240 Å. In the simulation, we constructed a perfect GaN matrix with a size of 50a×50a×50a containing 500,000 atoms. Then, based on the perfect GaN matrix, we constructed an imperfect GaN matrix with a vacancy defect rate of 10% and doped it with iron (Fe) atoms at a doping density of $5\times 10^{18}\;{cm}^{-3}$. To simulate the radiation of α-particles on the GaN matrix, we added two positively charged nitrogen atoms as α-particles above the system in the z-direction and gave the α-particles an initial velocity to shoot at the GaN matrix as PKA. To avoid channeling effects, a slight angle was added to the PKA incident direction [8]. In the MD simulation calculation, the particle trajectory was obtained by solving Newton’s equation. The most important factor was finding an appropriate energy relationship formula to describe the interaction between particles, usually represented by a potential function. Therefore, we used the Tersoff potential to describe the interaction between GaN particles [19,38], then used the Lennard-Jones (LJ) potential to describe the interaction forces between GaN and Fe atoms as well as α-particles [39,40], and finally smoothed the Tersoff and LJ potential with the Ziegler–Biersack–Littmark (ZBL) potential so that high-energy short-range interactions within the material could be better described [19,41]. See Table 1 for specific parameter settings.

**Figure 1:**
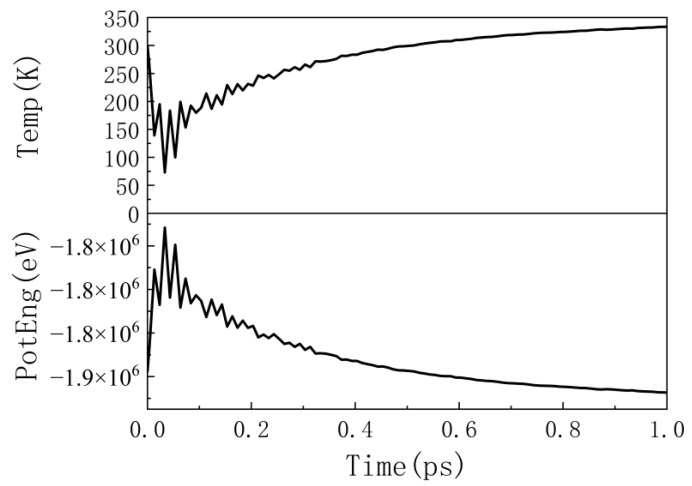
Temperature and potential energy of the simulation system during the relaxation stage as time functions.

Periodic boundary conditions were applied along the x- and y-directions for the simulation system, while fixed boundary conditions were used along the z-direction [20,42,43]. The entire simulation process can be divided into three stages. The first stage is the relaxation stage: we first relaxed the entire simulation system by conjugate gradient minimization to release the stress in the system [44]. Then we allowed the system to relax under the NVT ensemble for 10 ps, during which the system temperature was 300 K, and the time step (∆t) was set to 0.0001 ps until the system reached a stable state [45]. In this stage, the temperature and potential energy of the system can reach equilibrium, making the structure reach a metastable state, as shown in Figure 1.

**Figure 2:**
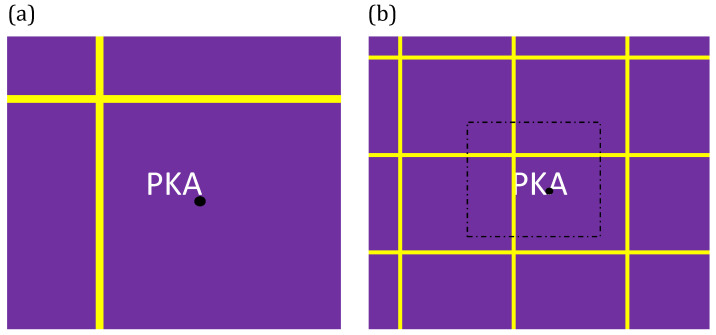
A schematic diagram of the two perpendicular constant-temperature walls: (**a**) the yellow area represents the constant-temperature walls; (**b**) constant-temperature walls under periodic boundary conditions.

In Figure 1, it can be observed that the temperature and potential energy change trends during the relaxation stage are opposite, and both eventually approach stability, indicating that the system has reached a stable state and can proceed to the next simulation step. The second stage is the collision stage: two perpendicular constant-temperature walls were used [8,45,46], as shown in Figure 2a. Figure 2b shows the view from the z-direction under periodic boundary conditions, with black dots representing the position of the incident α-particle.

The thickness of the constant-temperature walls was set to 2a, and the Langevin thermostat was used to maintain a constant temperature of 300 K for the atoms inside the walls so that excess kinetic energy introduced by PKA could be dissipated by the walls [47]. A fixed layer with a thickness of about 3a was selected as the bottom layer of the system to prevent the entire simulation system from drifting during the collision cascade process [18,21]. The remaining atoms were in the active region, and all atoms in the active region were restricted to adiabatic motion using the NVE ensemble [44,45]. In addition, to ensure that the atomic displacement in each time step did not exceed 0.1 Å, we used a variable time step (∆t) [44,47]. The final stage is the annealing stage: in this stage, the time step (∆t) was increased to 0.1 ps, and the system was annealed for a long time until there was no significant change in the total number of defects [19,45].

The Wigner–Seitz cell recognition method was used to identify defects caused by irradiation in the simulation system, such as vacancies, interstitials, and anti-site atoms [35,36]. A vacancy is a defect with no atom on a lattice site, while an interstitial defect is when two or more atoms are on a lattice site. An anti-site atom defect occurs when an atom of the wrong type occupies a lattice site. Defects within a cutoff distance of 1.5a were considered defect clusters [13], and the size of a cluster was defined as the number of internal defects. Different sizes of clusters were classified as small clusters (containing from one to six defects), medium-sized clusters (containing 6–30 defects), large clusters (containing more than 30 defects), and local amorphization (when a cluster contains more than 100 defects).

In this study, MD simulations were performed on GaN irradiated by α-particles. First, single α-particle irradiation on GaN was simulated, corresponding to an injection dose of 4 × 10^11^ ions/cm^2^. Two different energies were selected as the incident energy of the α-particle: 0.1 and 0.5 MeV. Then, multiple α-particles were simulated to accumulate irradiation damage in GaN, with 5 and 10 α-particles used for cumulative irradiation, corresponding to injection doses of 2 × 10^12^ and 4 × 10^12^ ions/cm^2^, respectively.

## 3. Results and Analysis

### 3.1. Simulation of Radiation Damage in GaN by Single α-Particle Irradiation

**Figure 3:**
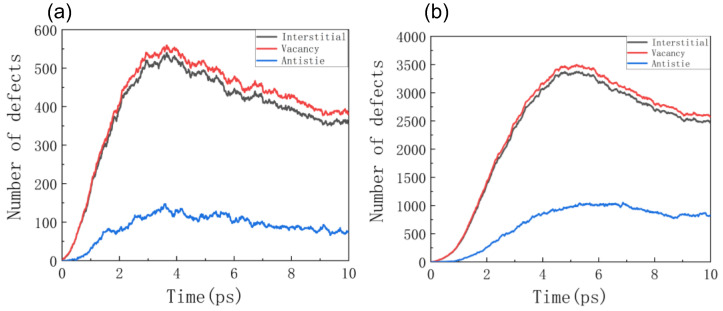
The relationship between the number of defects and time under the irradiation of α-particles with different energies: (**a**) 0.1 MeV; (**b**) 0.5 MeV.

The relationship between the number of defects caused by the irradiation of single α-particles with different energies and times was simulated by the MD method. Figure 3a shows the variation in the number of defects with time under 0.1 MeV α-particle irradiation. Figure 3b shows the variation in the number of defects with time under 0.5 MeV α-particle irradiation.

It can be seen from the figure that under irradiation with either energy level of α-particles, the number of defects increases first and then decreases to a steady state, with a peak value, namely, the displacement peak. This is because α-particles impact GaN, transfer energy to GaN through collisions, and cause displacement effects in the GaN system; the energy deposited in GaN is dissipated by the Langevin thermostat of the constant temperature wall, and GaN has a self-repairing ability, so the number of defects begins to decrease after reaching the peak value and eventually stabilizes. Compared with the displacement peak formed under 0.5 MeV irradiation, the time of the displacement peak formed under 0.1 MeV is slightly earlier. It can also be observed that the change trend of vacancy defects is the same as that of interstitial defects, and the number of vacancy defects slightly exceeds that of interstitial defects. This is because some interstitial defects with two or more atoms are formed during irradiation. Compared with the vacancy and interstitial defects, the figure shows little change in the peak and steady-state stages of anti-site defects, indicating that anti-site defects are more challenging to repair.

**Figure 4:**
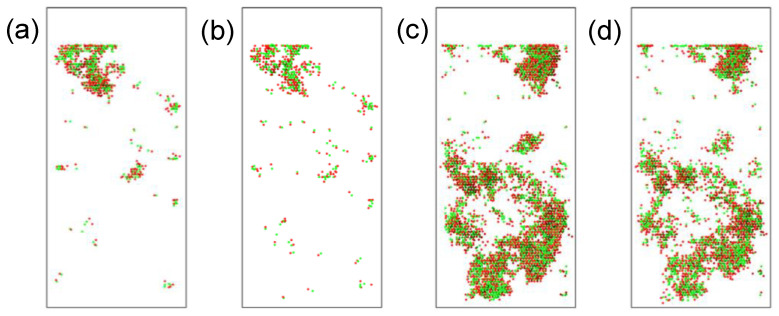
Defect distribution maps (in the x-direction): (**a**) peak stage at 0.1 MeV; (**b**) final stage at 0.1 MeV; (**c**) peak stage at 0.5 MeV; (**d**) final stage at 0.5. The green ball is the vacancy defect and the red ball is the interstitial defect.

The defect distribution in the x-direction is shown in Figure 4. Figure 4a,b show the defect distribution under 0.1 MeV, where (a) is the distribution at the displacement peak stage and (b) is the distribution at the final steady state. Figure 4c,d show the defect distribution under 0.5 MeV at the displacement peak stage and the steady-state stage, respectively. It can be seen from the figure that after long-time annealing, the number of defects is significantly reduced, and the defects caused by 0.1 MeV are primarily concentrated in shallow positions. In comparison, those caused by 0.5 MeV are concentrated in deep positions. In addition, it can be observed that local amorphization may occur in areas with high defect density. By comparing the results under 0.1 MeV and 0.5 MeV, the results show that the higher the energy level of α-particles, the more severe the damage caused by them.

To compare the self-recovery ability of GaN under different irradiation energies, we recorded its recombination efficiency in Table 2. As shown in Table 2, in GaN with a fixed size, under the irradiation of α-particles with different energies, the higher the energy is, the more severe the displacement damage caused and the more difficult it is for the material to self-repair. Under α-particle irradiation, we used the Wigner–Seitz cell recognition method to identify and analyze the type and quantity of each defect, as shown in Table 2.

The Ga-N (Ga) interstitial defect in Table 2 represents a vacancy containing both Ga and N atoms at the original Ga site, where (Ga) is the original Ga site, and Ga-N represents the Ga and N atoms. In the case of low-energy α-particle irradiation, the material has a stronger ability to repair Ga vacancies than N vacancies. In contrast, under high-energy α-particle irradiation, N vacancies have a stronger repair ability than Ga vacancies. In addition to interstitial defects containing two atoms, interstitial defects containing three and four atoms can also be produced under α-particle irradiation. Among the interstitial defects, Ga-N (Ga) and Ga-N (N) interstitial defects are easier to form, followed by Ga-Ga (Ga) and N-N (N) interstitial defects. Interstitial defects containing three atoms are relatively difficult to form, while interstitial defects containing four atoms are the most difficult to form and are not easily repaired by the material’s self-recovery ability once formed. Compared with 0.1 MeV irradiation, under 0.5 MeV irradiation, although the repair abilities of some types of defects increased and some decreased, the overall recombination efficiency decreased. This again indicates that the higher the energy of the incident α-particles, the more difficult it is to repair the displacement damage formed.

**Figure 5:**
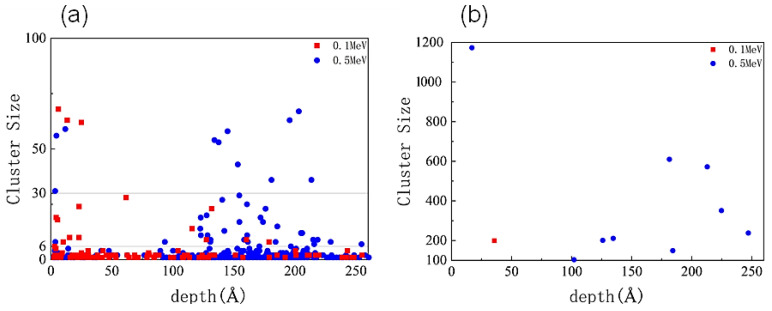
The size and distribution of defect clusters: (**a**) Number and location of large, medium, and small defect clusters; (**b**) Number and location of amorphous regions.

As shown in Figure 4, the defects formed after irradiation can also form defect clusters due to the aggregation of defects. When the defect clusters are too dense, they can form local amorphization. The presence of defect clusters and local amorphization in the material can seriously affect semiconductor materials’ mechanical and electrical properties. Figure 5a shows that more defect clusters were produced under 0.5 MeV α-particle irradiation, and the clusters were located deeper. More defect clusters produced under α-particle irradiation were small clusters, with more clusters concentrated within 125 Å under 0.1 MeV. Most positions under 0.5 MeV were at depths of more than 125 Å. Figure 5b shows the number and location of local amorphization under irradiation with different energies. Under irradiation with higher energy, more local amorphous regions will be produced within the crystal, and their positions will be deeper.

### 3.2. Radiation Damage Simulation of Multiple α-Particle Cumulative Irradiation on GaN

**Figure 6:**
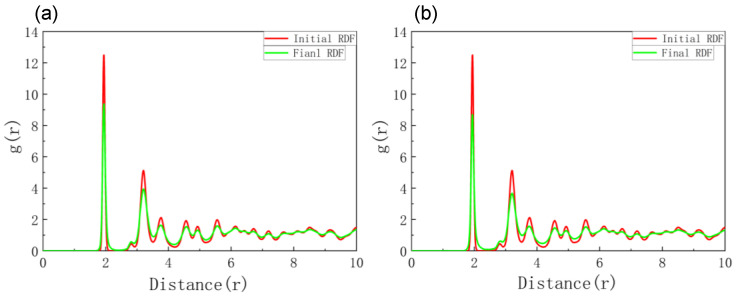
Radial distribution functions of GaN material under different dose rates: (**a**) accumulation of five injected α-particles; (**b**) accumulation of ten injected α-particles.

After simulating the single α-particle irradiation of the GaN matrix, we also studied the cumulative damage effects caused by multiple α-particle irradiations on GaN. In this case, the energy of the incident α-particles was set to 0.1 MeV, and the injection doses were 2 × 10^12^ and 4 × 10^12^ ions/cm^2^, corresponding to five and ten incident α-particles, respectively. Figure 6 shows the radial distribution function of the GaN material at two injection rates [48].

The red curve represents the radial distribution function in the initial state, while the green curve represents the radial distribution function in the final state. In the absence of α-particle irradiation, the number of first-nearest neighboring atoms is nearly 13, and the number of atoms from the second-nearest neighbor to the nth-nearest neighbor decreases slowly. At this time, the radial distribution function shows a long- and short-range order and is relatively ordered. After accumulating five α-particles, the number of first-nearest neighboring atoms in the GaN material decreased to about nine, as shown in Figure 6a, and the number of atoms from the second-nearest neighbor to the nth-nearest neighbor also decreased. Figure 6b shows that after accumulating ten α-particles, the number of first-nearest neighboring atoms in the GaN material also decreased to the value slightly exceeding nine, and the change in the number of atoms from the second-nearest neighbor follows the same pattern as in Figure 6a. At this time, regardless of the injection rate, the long-range order of the radial distribution function became disordered, indicating that under the cumulative irradiation of multiple α-particles, the internal amorphization of GaN material became increasingly severe.

**Figure 7:**
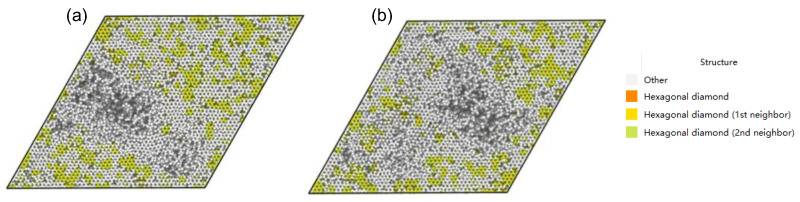
IDS surface topography of GaN materials in the z-direction under different injection rates: (**a**) accumulated injection of five α-particles; (**b**) accumulated injection of ten α-particles.

After accumulating irradiation, we further analyzed the damage inside the GaN material by examining the IDS damage morphology. Figure 7 shows the IDS surface topography in the z-direction, and Table 3 shows the percentage of each component in the IDS damage analysis.

As seen in Figure 7, a large hole appears on the surface of the GaN matrix, indicating that some surface atoms of the GaN matrix have been released from the confinement of the matrix lattice and sputtered out. The sputtering rates under different simulation conditions are listed in Table 4.

Figure 7a shows the IDS surface topography after accumulating five α-particle irradiations, and Figure 7b shows the IDS surface topography after accumulating ten α-particle irradiations. In both figures, a large hole appears on the surface of the GaN material due to the irradiation of the α-particles. Moreover, the entire hole has turned into an amorphous state on the IDS damage surface topography, indicating that the damage to the material has increased under the accumulated irradiation. Table 3 shows that there was already a 16.3% amorphous region in the initial state due to the imperfect GaN material with defects and doping used in this simulation. With the increased number of incident α-particles, the proportion of the amorphous region in the GaN matrix also increases. Under accumulated irradiation, the proportions of the hexagonal diamond, hexagonal diamond (1st neighbor), and hexagonal diamond (2nd neighbor) all decrease, with the hexagonal diamond decreasing the fastest. This suggests that the hexagonal diamond structure is the most susceptible to damage under the irradiation of multiple α-particles. Although the proportions of the hexagonal diamond (1st neighbor) and hexagonal diamond (2nd neighbor) also decrease under irradiation, they decrease slowly since the hexagonal diamond structure can transform into a hexagonal diamond (1st neighbor) or hexagonal diamond (2nd neighbor) after damage.

With increasing incident α-particle energy, the number of sputtered atoms and the sputtering rate also increases. Under cumulative irradiation, the sputtering rate increases significantly and increases with the number of injected particles. Therefore, large and deep damage holes will appear in the surface topography under cumulative injection.

Table 5 shows the types and quantities of defects under different cumulative irradiation conditions. It can be seen that with the increase in the number of α-particle injections, the number of each type of defect also increases. Compared with single α-particle injection, more types of damage are formed when multiple α-particles are injected, including gap defects containing more than five atoms. This indicates that the displacement damage of the GaN material is severe under cumulative irradiation and is not easily repaired by the material’s self-repairing ability. The table also shows that the number of N vacancies is always greater than that of Ga vacancies, indicating that the formation of N vacancies is more likely under α-particle irradiation. The number of vacancy defects is much greater than that of interstitial defects, which is consistent with the surface topography of IDS damage shown in Figure 7, where many atoms have been ejected from the matrix, leading to the formation of a large number of vacancy defects inside the matrix.

After comparing Table 2 and Table 5, it can be observed that under α-particle irradiation, the number of vacancy defects is higher than that of interstitial defects, and N vacancies are more likely to form. Those containing two atoms are more likely to form among the interstitial defects, while those containing more atoms are less likely to form. However, only under accumulated irradiation will interstitial defects containing more than five atoms form. Among the interstitial defects containing two atoms, Ga-N (Ga) and Ga-N (N) defects are more likely to form. To further understand the defect distribution and amorphization degree of GaN under accumulated irradiation.

**Figure 8:**
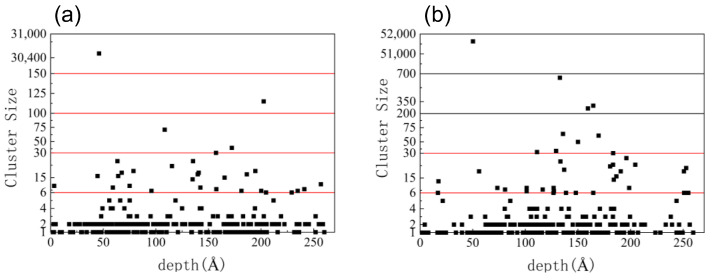
Size and distribution of defect clusters under different injection amounts: (**a**) accumulated injection of five α-particles; (**b**) accumulated injection of ten α-particles.

**Figure 9:**
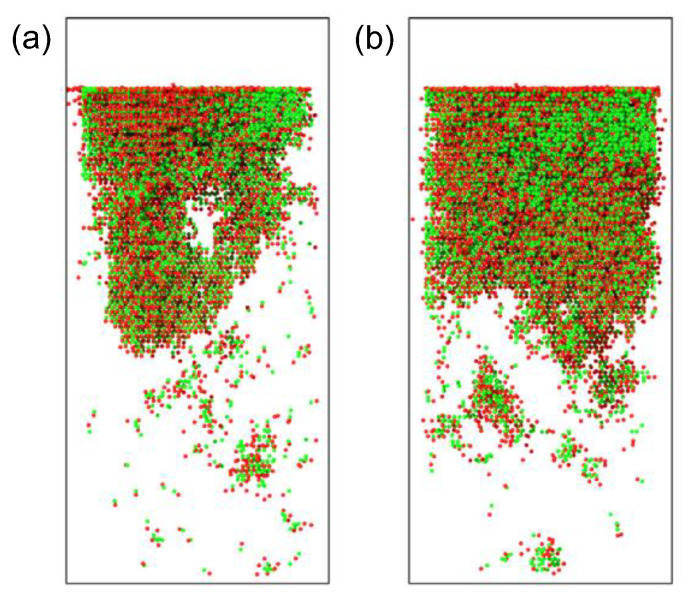
Distribution of defects under different injection amounts: (**a**) accumulated injection of five α-particles; (**b**) accumulated injection of ten α-particles. The green ball is the vacancy defect and the red ball is the interstitial defect.

Figure 8 shows the distribution of defect clusters. Figure 8a shows the distribution of defect clusters after accumulated irradiation of five α-particles, where a superlarge amorphous region containing more than 30,000 defects is formed. Figure 8b shows the distribution of defect clusters after accumulated irradiation of ten α-particles. A superlarge amorphous region containing more than 50,000 defects and several small amorphous regions containing 200–700 defects are formed. Under accumulated irradiation, most defect clusters are small or medium-sized, while the number of large clusters increases with the number of injected particles. At the same time, the number and size of amorphous regions also increase with the number of injected particles, as shown in Figure 9, where the amorphous region in Figure 9b is larger than that in Figure 9a. Furthermore, it can be observed that the amorphous region produced under the injection of five α-particles in Figure 9a is not a whole piece, but composed of several parts. The amorphous region produced by injection of ten α-particles in Figure 9b is a single block. This indicates that, at low dose injection, amorphous regions combine to produce large volume damage, while at high dose injection, large volume damage will be directly caused. At higher doses of cumulative injection, greater damage can appear further down.

## 4. Conclusions

This paper studied the displacement damage effect of α-particles on GaN material using the MD simulation method. The differences in damage between single α-particle irradiation at different energies and multiple α-particle injections were discussed. This paper mainly studied and compared the quantity, type, and distribution of defects, degree of amorphization, and repairability. The results obtained made it possible to draw the following conclusions. Under single α-particle irradiation, the displacement damage effect increases with increasing irradiation energy. N-vacancy defects are easier to form than Ga-vacancy defects, indicating that N atoms are more likely to break free from the lattice sites under irradiation. The self-repair ability of the GaN material decreases as irradiation energy increases. Defect recombination rate decreased from 32% to 26%. In addition, there is little difference in the repairability of vacancy and interstitial defects in GaN material. The defects caused by 0.1 MeV irradiation are more concentrated within 125 Å, while the defects caused by 0.5 MeV irradiation are mostly outside 125 Å. Under single α-particle irradiation, the size and depth of defect clusters increase with increasing energy, and at higher energy injection, deeper locations form larger defect clusters. In the simulation of multiple α-particle injections, the amorphous regions become larger and more numerous with the increase in injected atoms. The amorphous region increased from 16.3% to 27.3% and then to 31.9%. The sputtering rate also increases with the number of injected particles, which increased to 0.512% and 0.638%. For the study of the mechanism of heavy ion irradiation material, especially the study of latent track, a variety of models have been proposed, such as the Coulomb explosion model, thermal peak model and exciton model, which can be used to simulate and analyze the damage caused by heavy ion irradiation. In addition, experimental verification can be added to combine the microscopic parameters of molecular dynamics with the macroscopic characteristics of future devices.

## Figures and Tables

**Table 1:** Set specific parameters.

Parameter	Value	Parameter	Value
Lattice constant	a = b = 3.216 Å c = 5.240 Å	Atomic number	500,000
Defect rate	10%	Doping rate	5 × 10^18^ cm^−3^
Crystal size	50a × 50a × 50a	Temperature	300 K
Incident energy	0.1 MeV 0.5 MeV	Injected dose	4 × 10^11^ ions/cm^2^ 2 × 10^12^ ions/cm^2^ 4 × 10^12^ ions/cm^2^

**Table 2:** Classification and quantity statistics of defects.

			0.1 MeV			0.5 MeV		
Defect Classification			Quantity	Repair Capability (%)	Recombination Efficiency (%)	Quantity	Repair Capability (%)	Recombination Efficiency (%)
Vacancy		Ga vacancy	166	41.34	31.31	1276	22.95	26.5
		N vacancy	217	21.38		1293	29.8	
Interstitial defect	Two atoms	Ga-N (Ga)	194	35.25	33.52	1116	26.86	26.92
		Ga-N (N)	120			1075		
		Ga-Ga (Ga)	22			92		
		Ga-Ga (N)	2			8		
		N-N (Ga)	0			13		
		N-N (N)	20			90		
	Three atoms	N-N-N (N)	0	18.19		1	28.57	
		Ga-Ga-N (Ga)	2			24		
		Ga-Ga-N (N)	1			9		
		Ga-N-N (Ga)	3			22		
		Ga-N-N (N)	3			14		
	Four atoms	Ga-Ga-N-N (N)	0	/		1	/	
		Ga-Ga-Ga-N (Ga)	0			1		

**Table 3:** Proportions of different components in IDS damage analysis.

Structure	Initial State (%)	Five α-Particles (%)	Ten α-Particles (%)
Other	16.3	27.3	31.9
Hexagonal diamond	18.1	14.0	12.8
Hexagonal diamond (1st neighbor)	29.6	24.7	23.1
Hexagonal diamond (2nd neighbor)	36.1	34.0	32.2

**Table 4:** Sputtering yield under different simulation conditions.

Mode	Number of Sputtered Atoms	Sputtering Yield (%)
Energy of 0.1 MeV	26	0.0058
Energy of 0.5 MeV	33	0.0073
Accumulation of five α-particles	2302	0.512
Accumulation of ten α-particles	2871	0.638

**Table 5:** Defect classification and quantity statistics under different cumulative irradiations.

			Five α-Particles	Ten α-Particles
Defect Type			Quantity	Quantity
Vacancy		Ga vacancy	5669	12,093
		N vacancy	11,473	16,780
Interstitial	Two atoms	Ga-N (Ga)	8974	12,803
		Ga-N (N)	3383	8144
		Ga-Ga (Ga)	323	712
		Ga-Ga (N)	254	470
		N-N (Ga)	234	426
		N-N (N)	153	399
	Three atoms	N-N-N (N)	9	16
		Ga-Ga-N (Ga)	384	623
		Ga-Ga-N (N)	94	159
		Ga-N-N (Ga)	125	291
		Ga-N-N (N)	101	313
	Four atoms		116	256
	Five atoms or more		232	324

## Data Availability

Not applicable.
